# Normal Ovariotomy

**Published:** 1873-04

**Authors:** Robert Battey

**Affiliations:** Rome, Georgia


ATLANTA

### MEDICAL AND SURGICAL JOURNAL.

VOL. XI.—APRIL, 1873.—NO. 1.

---

### ORIGINAL COMMUNICATIONS.

---

### NORMAL OVARIOTOMY.

BY ROBERT BATTEY, M.D., ROME, GEORGIA.

(*Read Before the Georgia Medical Association, Atlanta, Georgia, April,* 1873.)

Since our last convocation, in the city of Columbus, I have felt it to be my duty to enter the domain of surgery, and carve out for myself a new pathway through consecrated ground, upon which the foot of man has not dared wittingly to tread. I doubt not it is known to you all that I have invaded the hidden recesses of the female organism and snatched from its appointed seat a glandular body, whose mysterious and wonderful functions are of the highest interest to the human race,—nay, an organ endowed with functions, the integrity of which determines the very existence of the race itself. For having done this, I trust I shall not be *assassinated*, neither in my carriage at home, nor in this hall, nor yet upon the streets of your orderly city. Those of you, my brethren, who know me personally, I hope will scarcely need the assurance that I have not taken this step forward without mature and deliberate thought. Whatever may be your opinions of the wisdom of my course, I trust you will see in it evidences of a heart not devoid of human sympathy—of a mind not shirking professional labor—of a hand not fearing to lift itself when duty calls.

However pure may have been the motives actuating me,—however cogent may have appeared, to my own mind, the reasons which have impelled me,—I must of necessity stand before the bar of the medical world, and submit myself to its just judgment. It becomes me, too, to appear before you, my brethren and my peers, to answer for myself.

And *first*, for the facts. These have already been spread before the profession through the pages of the ATLANTA MEDICAL AND SURGICAL JOURNAL, which has an extended circulation among the members of this body, as well as in the profession generally of this and the surrounding States. These facts have likewise been published in pamphlet form, and widely distributed to the medical press and medical men, both in this country and in Europe. This statement of facts has probably been read by the great majority of the members present, and I will gladly save you the infliction of a rehearsal of the pamphlet, which I now present you as the basis of the remarks I have to make.

Allow me here to correct an error which has inadvertently crept into my report, in stating the *age* of my patient, Miss Julia. She was twenty-three years old when she first came under my professional charge, and was *thirty* at the time of the operation.

In continuation of the history of my case from the publication of the pamphlet down to the present, we shall not consume much time.

HISTORY CONTINUED.

*Thirty-fourth Day.*—She feels fullness about the head and pain in the back, which reminded her of the premonition of one of her old attacks. She was put under the influence of bromide potassium, and the nervous symptoms soon passed off.

*Forty-second Day.*—Complains again of pain in the left iliac fossa, and had *metrostaxis.*

*Forty-third Day.*—Pain relieved by blister; metrostaxis continues.

*Forty-fourth Day.*—Uterine flow has ceased.

*January* 1, 1873.—She has had a uterine flow for four days, without headache or any material nervous disturbance. This is the third attack of metrostaxis since the operation.

*January* 24.—Complains a little of soreness and pain in the left iliac region.

*February* 1.—Metrostaxis for one day.

*February* 5.—Complained a little on yesterday and to-day of headache and pain in the back; ordered a purgative and bromide, which soon relieved the symptoms.

*February* 7.—Nervous symptoms are all gone. She is fattening up decidedly, and rapidly gaining strength. *She is ten pounds heavier than ever before in her life.* She has several times walked a mile, spent the day visiting, and walked home again in the afternoou. She appears bright and cheerful and happy, and says her nervous attacks are trivial in comparison to those suffered previous to the operation. She has passed the winter without cough or any chest symptoms, and without rheumatism, except some trivial aching in the wrist and elbow.

*February* 15.—Attacked with bilious dysentery, ushered in by rigor and fever, and attended with a very free metrostaxis—freer than she has ever experienced before. The metrostaxis contintinued for sixty hours, and the dysentery for several days.

*March* 22.—She is visiting her friends from house to house, attends her church regularly upon the Sabbath, and shops upon the street. She walks a mile with ease.

As far as I am able to discover, *she has lost nothing of value whatever in consequence of the operation;* and the patient herself, who was duly informed, prior to the operation, of *all* its possible consequences, assures me that in no respect does she find herself different from her former self, excepting in the absence of her previous sufferings, and the wonderful improvement in her health and spirits.

*April* 7.—Condition the same.

Having removed by incision the ovaries of my patient, I very naturally felt that I had done an *ovariotomy;* but when I attempted a comparison with the recognized operation of our friends, the ovariotomists, I found at once striking differences, both in the thing done and the purpose of its execution. It seemed to me well, under the circumstances, to recognize at once these differences, by adopting a new and distinguishing appellation. Reflecting a little upon the matter, it appeared to me that it was *I* who had really and truly done an ovariotomy, rather than Dr. Ephraim McDowell, as I understand the *rule,* or law, or principle, which governs medical nomenclature in such cases. My proceeding, therefore, seemed to me to be an ovariotomy, *according to an established law, rule, or principle.* By this custom, law, rule, or principle, I felt that my operation was *regular* ovariotomy, and McDowell's irregular. As my proceeding removed the organ prior to its degeneracy by disease, it seemed to me an ovariotomy which *related to rudiments or elements.* And moreover, if it be possible to say that an operation of such nature is in any sense *square*, then mine is the square ovariotomy. But all this was a little too complex. I desired, if possible, to express my thought on the point in a single word, and, referring the matter to Webster, I readily selected the word *normal* as expressing the qualifications:—first, "according to a square or rule;" second, "regular,—according to an established law, rule, or principle;" and third, "relating to rudiments or elements."

Of course, I could not conceive that an operation for the removal of the normal ovaries could in any sense be a *natural* ovariotomy.

In considering the propriety of the operation of normal ovariotomy in the case recited, it will be pertinent to inquire:

1. Was there rational ground for the belief that the change of life would cure my patient of her sore malady?

2. Was there rational ground for the belief that removal of the ovaries would bring about the change of life?

3. Was it justifiable to effect a cure at the sacrifice of her ovaries?

To the first of these questions, as it seems to me, there can be but one answer. My patient was suffering with the perturbing influences of an unrelieved menstrual molimen. The change of life is understood to include both the cessation of the menses and the cessation of the menstrual impulse. The removal of the menstrual molimen—the exciting cause of the perturbation—would logically remove the effect with the cause. Upon this point allow me to quote a single authority. Dr. Tilt says:

"With respect, however, to women who had been suffering for many years from intractable chronic affections that had baffled our best efforts to bring about recovery, the results of cessation are in general eminently satisfactory. We are, most of us, in the habit of telling our patients that they will be certainly cured by the change of life; and although this promise is often given to keep up the patient's hope, rather than as the result of a well-grounded prognosis, still it is surprising how frequently the prophecy proves true. This remark particularly applies to *ovarian congestion* and subacute inflammation, and to most *chronic diseases of the womb.*

"I have notes of some forty patients, who were for many years before the menopause confined to the bed or the sofa by chronic uterine inflammation, who made marvelous recoveries very soon after the change was effected, and who are once more actively engaged in those pleasures and duties of society from which they had been divorced for ten or fifteen years. Out of many similar cases, in which recovery was not thus rapid and perfect, I can not call to my recollection a single instance in which great improvement was not obtained. I have also ascertained from twenty-six women, who had ceased to menstruate, that they were no longer troubled by habitual leucorrhœa, and doubtless many suffer for years from unrecognized uterine affections, which are at least completely cured by the change of life. Prolapsus of the womb was cured in three cases; thirty-five women no longer suffered from uterine deviations, though they still existed; in four cases, varicose veins had gone down; in twenty-four, piles had disappeared; and in eight other cases, they had ceased to bleed. Fifty-three women spoke of the great additional strength obtained, and of the abatement of their liability to dyspepsia. *Ganglionic affections* then often lose their gravity, and become less frequent; and the same remark applies to almost all *cerebro-spinal* affections, even to the most formidable—for Ésquirol has seen many women remain maniacal so long as menstruation lasted, who immediately and spontaneously recovered after the menopause."*

Who is there among you, my brethren, who, having had some years of observation, can not recall few or more cases to corroborate these observations of Dr. Tilt?

If the ovular theory of menstruation is to be accepted as an ultimate fact, the answer to my second question becomes as easy and as logical as the first. If the menstrual nisus be simply the result of ovulation, then the cessation of ovulation, the cause, necessitates the cessation of the effect of that cause, whether this cessation occur by reason of atrophy of the ovaries by age or their removal by the knife. It seems to me, there-

* Change of Life. Philadelphia, 1871. p. 20.

fore, that the question to be considered is, *Are there reasonable grounds for the acceptance of the ovular theory of menstruation?*

I do not propose to undertake the discussion of this theory. I will not consume your time and weary your patience with the unnecessary details, but shall content myself by citing such authoritative opinions as are to be found upon my own shelves, and which, I shall contend, fully warrant me in accepting the theory as an ultimate fact so far as my duties to my patient are concerned.

Upon the subject of menstruation, Dr. Charles West uses the following language:

"The ovaries are the grand organs of sexual activity in the female; and during the whole time that sexual life continues, they are employed in bringing ova to maturity, and then in extruding them at certain periods when they have attained a state of fitness for further development, if subjected to the fecundating influence of the semen. Accompanying this internal process, *the consequence* and the evidence of the local congestion which attends it, we observe a periodical discharge of blood, constituting menstruation."*

Dr. Meigs says:

"I adopt the notion that the act of menstruation, rigorously construed, consists in the periodical maturation and deposit of an ovulum—of which act the flowing of the menstrual blood is but the outward and visible sign."†

Montgomery says:

"In fact, it appears that each menstrual nisus may be regarded as an abortive effort at reproduction, and the elimination of the discharge itself, simply as the necessary disposal of a certain amount of blood, which, had conception taken place, would have been devoted to the support and maturation of the verified ovum; or, to use the words of Dr. Power, 'a woman menstruates because she does not conceive;' and it has been happily said, by Dr. Tyler Smith, that menstruation may be considered as the first act of human parturition; it is, as it were, the parturition of the ovule, while labor is the parturition of the matured ovum."‡

* Lectures on the Diseases of Women. Philadelphia, 1857. p. 20.
† Woman and Her Diseases. Philadelphia, 1854. p. 419.
‡ Signs and Symptoms of Pregnancy. Philadelphia, 1857. p. 387.

Tyler Smith says "the *cause* of menstruation must be referred to the ovaria. We may look, then, to the ovaria for the exciting cause of menstruation, and this function is evidently subsidiary to that of ovulation."*

Dr. Meigs says:

"Menstruation, therefore, strictly interpreted, is ovulation, and the sanguineous discharge, that is vulgarly considered as the principal point, is far less principal than the ovarian ovulation—of which, indeed, it is only the outward mark or symptom. The true doctrine was that of a local plethora, or, in other words, a state of periodical hyperæmia of the reproductive organs; and now that doctrine is not only established, but is made plain to the understanding—for the periodical paroxysm of stromatic force, that hurriedly concludes the ripening of the most perfect ova, establishes the affluxion that fills the capillaries of the reproductive organs, and engorges them, or renders them hyperæmic to the point of causing the monthly hemorrhage by which the hyperæmia is removed, leaving behind it no trace of indisposition.

"When Percival Pott, the illustrious surgeon, removed the ovaria of his patient, under an operation for hernia, he took away with them the power of menstruation. There are numerous examples of females who did never menstruate, owing to the absence of the ovaries.

"Patients suffering with chronical maladies, attended with protracted amenorrhœa, exhibit, in the ovarian stroma, no vestiges of the Graafian vesicles. I lately examined the ovaria of a girl who died after some eighteen months of severe chronical ailments, during which she did not menstruate. Those ailments had no primary connection at all with any state of the reproductive organs; yet, upon carefully examining the ovarian stroma of both the ovaries, it was found to be a compact, whitish tissue, very similar to that which we observe in women long past the change of life. No trace of the ovarian vesicle existed in either of them. It is generally so."†

Scanzoni says:

"Under the name of menstruation, we commonly understand

* Lectures on Obstetrics. New York, 1858.

† Obstetrics: The Science and the Art. Philadelphia. pp. 146, 152, 156, 167.

a series of phenomena manifested in the female organism, and having for its first cause the periodic ovulation, which takes place in a Graafian vesicle."*

Bedford says:

"What, pray, are these organs? They are the *ovaries*—the essential and only organs of generation, strictly so-called, in the female. The development of the ovaries occurs at the period of puberty, and then it is that their physiological action commences. At this time you will observe, on the surface of these bodies, the Graafian vesicle—this latter containing the ovule, which, I have told you, escapes ordinarily with the menstrual blood. As these ovules on the surface become matured, the ovary itself forms the center of a sanguineous afflux, a veritable congestion, in which the fallopian tubes and uterus participate. This congestion results in the escape of mucus and of blood, which pass from the uterus through the os tincæ into the vagina, and thence externally; and this is menstruation."†

Bennett says:

"The researches to which I refer prove, in the *most satisfactory and conclusive manner*, that menstruation is intimately connected with the evolution from the ovary of matured ova, which takes place periodically in the virgin as well as in the married female. In the human female the maturation and evolution of ova occur at frequent intervals, and are remarked by the exudation from the uterine cavity of a greater or less quantity of blood."‡

Tilt says:

"Nevertheless, as we positively know that the ovaries *rule supreme* over menstruation, and that they cause many diseases of women during the period of woman's greatest reproductive energy, it is fair to suppose that they aggravate and delay the cure of the most common uterine diseases, when ovarian irritation is no longer relieved by an habitual menstrual flow."§

Our own Sims says:

"It must be admitted, however, that menstruation is a sign

* The Diseases of the Sexual Organs of Women. New York. Fourth American Edition. p. 314.

† Diseases of Women and Children. New York, 1856. p. 233.

‡ Bennett on the Uterus. Philadelphia, 1860. p. 51.

§ Change of Life. Philadelphia, 1871. p. 273.

of ovulation, the one taking place when the other begins, and ceasing when it stops."*

Thomas says:

"That the discharge of blood, which occurring at monthly periods constitutes menstruation, is a true hemorrhage dependent upon the process of ovulation, is now regarded as a settled fact by most progressive physiologists. In accordance with a law of nature, which we recognize in its effects, but can not explain, once in every twenty-eight days, one or more ovules in each ovary burst their envelopes, and, entering the fallopian tubes, pass downward to the uterus. This eruption of ovules produces in the ovaries congestion and nervous exaltation, which continue until the process is completed."†

Byford says:

"Several conditions are necessary to the healthy performance of menstruation,—

"1. The ovaria must be present, and sufficiently healthy to produce ova.

"2. The uterus must be sufficiently perfect, anatomically and physiologically, to be the medium of this elimination.

"3. A certain, but not as yet very well-defined, state of the blood and nervous system.

"I do not think that these are all the conditions necessary to perfect menstruation; *they are the obvious and undoubted ones.* The physiological chain of circumstances that give rise to menstruation may be given thus: The organs concerned being fully developed, the blood and nervous system matured to a certain degree, an ovum is produced, and during the time it is being matured and cast off from the ovary, all the organs of generation are intensely congested by the increased energy of the capillary circulation; the congestion and stress of blood upon the delicate capillaries of the mucous membrane of the uterus become so great that the walls of some of these vessels are ruptured, and an effusion of blood takes place in the cavity of the uterus, which, finding its way out of the vagina, is called menstruation. *If ovulation does not take place, the congestion does not occur, and in the absence of the congestion, there is no effusion.*"‡

* Clinical Notes on Uterine Surgery. New York, 1866. p. 40.

† Diseases of Women. Philadelphia, 1869. p. 493.

‡ Medical and Surgical Treatment of Women. Philadelphia, 1867. p. 67.

Need I say more on this point? Was there rational ground for the belief that removal of the ovaries would bring about the change of life? Judge ye.

I can not, however, leave this branch of the subject without calling your attention to certain facts and observations which might seem to you to somewhat obscure the clear and convincing evidences of ovular menstruation, and I shall endeavor to satisfy you, if I may be able to do so, that it is an obscurity only in seeming, not in reality.

I quote, briefly, from Dr. Atlee's recent work upon "Ovarian Tumors," at page 35 and onward:

"CASE IV.— * * * April 17th, 1854, I operated on Mrs. J. C., of Baltimore, * * * removing both ovaries. She was thirty-five years old; had first menstruated at the age of twelve years, was married at the age of eighteen, and had six children, the youngest being four years old. * * * She nursed all her children, and while nursing she menstruated regularly, commencing at the expiration of five months after parturition. She continued to be very regular after the birth of her last child, up to the period of the operation. * * * After the operation, the same evening, the menses appeared, and continued the usual number of days. For several years Mrs. C. wrote to me on every anniversaiy of the day of the operation, always assuring me that menstruation was perfectly regular. Being on a visit to Baltimore, in December, 1866, I saw her, and she informed me that she had menstruated as regularly as ever up to May, 1864, when the menses ceased for one year, and again returned in May, 1865, for the last time."

"CASE V.— * * * April 25th, 1855, I removed both ovaries from Miss K. N., of Baltimore. * * * She was nineteen years old; she first menstruated before her thirteenth year of age, and was regular afterward. * * * Six months after the operation I saw the patient in Baltimore, where she had just issued her wedding cards. She had no *red menstruation* since the operation, but she experienced the usual sensations in her head and back at regular monthly intervals, accompanied with *white* discharge at those times. She married, made a visit to Europe, and after her return I learned through her mother that the monthly discharge continued, and that the sexual feelings were normal."

"CASE VI.— * * * October 16th, 1867, I extirpated the left ovary of Mrs. J. C. * * * She was twenty-seven years old; had been married four years, but had not conceived. After slight irregularity in menstruation, she had first noticed the tumor in May, 1857. * * * The right ovary was examined and pronounced healthy.

"July 8th, 1861, Mrs. C. called to see me, having an ovarian tumor in the right side, which had existed for about six weeks, and was rapidly enlarging. Menstruation was regular. October 16th, 1864, she called again, and reported herself perfectly regular ever since. November 11th, 1864, I removed the right ovary. * * * The left side of the uterus was examined at the time, and found to be perfectly truncated, being short of every kind of appendage."

Her physician writes Dr. Atlee on December 8th, 1870:

"She informed me that from the time of the first operation up to the second, she menstruated regularly; since the last operation she has been regular, and is to-day. *In fact, she is at this time menstruating.* When I say regular, I mean, of course, *that in its fullest sense;* she is regular as to *time, quantity, quality, etc.*, and free from any abnormal symptoms."

"CASE VII.—Both ovaries removed,—one in 1846, by Dr. Charles Clay, of Manchester, England, the other by myself, in 1861; menstruation always regular. * * * During the whole of the above period, notwithstanding one ovary had been extirpated, and the other ovary was extensively diseased, menstruation not only returned regularly, but conception took place, gestation was matured in spite of repeated tappings, and a living, healthy child born! And yet more, menstruation continued to recur regularly afterward, as she writes from Wisconsin, October 24th, 1863: '*Courses all right every month*'!"

On the 23d September, 1865, Dr. H. R. Storer, of Boston, operated upon Sarah A. Colcord, of Malden, unmarried, aged forty-seven, and removed the *uterus as well as both ovaries.* Dr. Storer says:

"I now (November 9th) made the first vaginal examination since the operation, and found the cervix reduced to a mere nodule, button-shaped, and much smaller than I expected to find. From the date of the operation until October 11th, eighteen days subsequently, and twenty-six days after the last appearance of the catamenia, there was no discharge whatever from the vagina. There now ocurred a sanguineous effusion, attended by feelings of lassitude, backache, etc., lasting thirty hours, and being an evident attempt at the reëstablishment of menstruation,—a very curious circumstance, and of great physiological interest, when it is recollected that the uterus and both ovaries had been removed. The ensuing period has been passed without its recurrence."*

From Peaslee's new work on "Ovarion Tumors," page 527, I quote another case of Dr. H. R. Storer's:

Mrs. Durham, aged forty-three years, the mother of six children. Menstruation had been regular down to within two months, and was now suppressed, as was supposed, by pregnancy. The symptoms became urgent, and November 20, 1867, both ovaries were removed. In the present instance, the menses had been absent for two months, and yet reappeared subsequently to the operation, although the ovaries had both been removed, and the major part of the fallopian tubes also."

In the *American Journal of Medical Science*, for January, 1868, page 81, Dr. Storer says:

"I removed both ovaries, a year since, from a patient in Brookline, Mrs. Mathews. In this case I deviated from the usual method of dealing with the pedicle, in that I did not divide the pedicle, as is usually done, but carefully dissected away the fallopian tubes, throughout their whole length, from the surrounding masses, preserving them intact, and then closed the peritoneal wounds along their entire course by metallic wires. * * * The ovaries were entirely removed, and yet the patient has had during the supervening period, quite regularly, a sanguineous discharge, without evidence of uterine disease, and which hæmostatics, generally and locally applied. have failed to check or prevent."

Dr. Peaslee cites from his own practice and others, twelve or thirteen other cases, in most of which the sanguineous discharge occurred but once or twice, and goes on to remark: "Unquestionably, therefore, a sanguineous flow occurs *per vaginam*, in exceptional cases, after double ovariotomy; this obtaining only once or several times, at regular or irregular intervals, and very

* American Journal of Medical Science, January, 1866. p. 119.

soon or a considerable length of time after the operation. But can such a flow be appropriately termed menstruation?

"If, for the moment, we set aside all the generally received ideas of menstruation as depending on ovulation, I suppose no one would accept as menstruation the flow which occurs but once, and in the first week after the operation, or indeed at any point of time thereafter. Such a hemorrhage is a mere uterine epistaxis, or metrostaxis, as Mr. Wells has appropriately named it, and may arise from the body of the uterus alone, or the cervical canal, or both, at the same time.

"Thus we have in literature six cases of apparent menstruation after double ovariotomy. Was it really such?

"If we define menstruation to be the sanguineous flow which is produced by ovulation, or which merely accompanies the ovulation, of course the term is here inapplicable, since, in the absence of the ovaries, ovulation is impossible. But there is no proof, nor any probability, that ovulation produces true menstruation. Certainly, ovulation and even parturition occurs in some women who have never menstruated. Menstruation is, therefore, an accidental and an incidental, rather than an essential function, and it has no analogue in most of the lower animals. In itself considered, it is merely a flow of blood at stated periods from the interior of the uterus, irrespective of its connections or causation. But, in its scientific acceptation, it has always been restricted to the flow from the cavity of the uterus and the fallopian tubes, which returns once a month to a healthy, non-pregmant woman of the child-bearing age. More recently, it has been found also that ovulation occurs especially, but not exclusively, at the same time; and physiologists are, therefore, obliged to associate this idea also with menstruation, as the before-mentioned characteristics always have been, and we must have some term to express precisely these ideas, and no more nor less. We must, therefore, cease to use the term in this sense, and substitute another, or retain it in this sense alone. In other words, if an exceptional uterine flow, in circumstances such that ovulation is impossible, be called menstruation, the same term must not be applied to the flow which physiologically accompanies ovulation. No one, I suppose, proposes to relinquish the term in the latter circumstances; it must, therefore, not be applied in the former, but a new term must be used, and metrostaxis is unobjectionable. Metrostaxis may occur at any time, and does occur under very diverse circumstances, from any part of the uterine cavity, or the cervical canal, in a congested state of the internal vessels of the non-pregnant uterus; and may occur from the cervical canal alone even during pregnancy, or after the removal of the ovaries and all of the uterus except the cervix, as in Koeberle's case.* The flow may also, though very rarely, become quite regular, as we have just seen in cases in which both ovaries have been removed. *But all this is mere metrostaxis, and not menstruation,* and can not be cited to disprove any theory of the causation of true menstruation; and no such theory, therefore, need here be discussed.

"Thus double ovariotomy, as a rule, is not followed by any loss of the special characteristics of woman,—the only decided physiological change being a *final cessation of menstruation, as well as of ovulation.*"

Dr. Atlee reasons this point as follows:

"Even when both ovaries are diseased, the menses may appear without any apparent derangement; and this may be accounted for on the supposition, that in the diseased ovary there may still exist Graafian vesicles in a normal condition. The removal of one ovary does not necessarily prevent ovulation nor impregnation,—provided the remaining ovary, though diseased, contains Graafian vesicles in a healthy condition. And what is more remarkable, as a physiological fact, the removal of both ovaria is sometimes followed by a *regular red discharge*, even for years, and until it is arrested at the usual climacteric period. Perhaps this may be the result of a habit, or habitual molimen, just as a eunuch, whose testicles have not been removed until puberty has been established, may have both erection and the ejaculation of a fluid."

In the *New York Journal of Medicine*, for 1844, is to be found "a remarkable case in the practice of Dr. LeConte, of Georgia, in which a negress *seventy years of age*, in whom the menses had been absent more than twenty years, was treated by thunder and lightning from the Almighty's battery, one charge from which brought on the menstrual flow, which continued regularly for the two succeeding years."†

* And even after removal of ovaries and uterus, except a small segment of the cervix, as in the case before cited, of Sarah A. Colcord.

† Journal Gynæcological Society, September, 1872. p. 238.

In the *London Lancet* (reprint) of 1861, page 190, is reported the case of a widow lady, aged *seventy-four:* "She has had for the last six months a periodical hemorrhage from the uterus, returning every three weeks or so, the discharge exactly resembling, she says, what it was when she was young. She complains of nothing but gradually increasing weakness. Has long-standing heart disease."

In the same connection, Dr. O. N. Royle, F.R.C.S., reports three other analogous cases: "One in private practice, who during the last year of her life was the subject of a regular periodical discharge, resembling in appearance the catamenial secretion of her youth. She died a few months since, in her *sixty-seventh year*, of anasarca, the result of long-continued disease of the heart and liver. The two other patients were union cases. One died last year in the infirmary here, to which I am surgeon, at the good old age of *ninety-three*, in a perfect state of health up to the day of her death, with this exception (the catamenia), save an obscure fluctuation which she occasionally complained of in the abdomen, at the bifurcation of the aorta, a few months previous. The third party is still living, and in her *sixty-seventh year*. For the last fifteen months she had ascites, and during that period I have had occasion to tap her six times. Since the first tapping, however, she has never experienced any return of her monthly sickness, which regularly occurred three months previously."

Bear with me while I cite from my own practice another case which, I can not but think, has an important bearing upon this question.

Some years ago I was summoned to visit Mrs. S., a lady of sixty years. As she entered her parlor, and extended the hand to me in her usual cordial manner, she remarked:

"Doctor, I know that you will think me an old fool to send for you! But I must tell you. I am not sick; I never enjoyed better health in my life. I have neither pain nor ache anywhere; I have good appetite, good digestion, and sleep well; I never was stronger; I run up and down stairs all day long, and attend regularly to my own household affairs. I changed life at forty-five; had some little trouble, but not very much, and have had good health ever since. But of late I have become *regular* again just like I was when a girl. I feared it might not be quite right, and thought I would like to talk with you about it."

"I think you did well, Madam, to send for me. Tell me, how long has this been?"

"Nearly a year, Doctor."

"How often do you come so?"

"I am perfectly regular, Doctor, like I used to be."

"And how long does it last, and how about the quantity?"

"Why, four or five days, Doctor; and just like it used to be."

"Does it weaken you at all?"

"No,—not a bit."

"Is the discharge offensive?"

"Not at all."

"You perceive nothing wrong about you at all, excepting this return of the menses?"

"No,—nothing."

"Well, Madam, I shall not laugh at you for calling me. I think you were right in asking advice; but I do not think it quite right for you to resume your youthful functions again in this way. I fear something is wrong, and I must see, I must know, what it is."

"Very well, Doctor, as you think best."

A careful exploration of this case revealed a hard, somewhat nodular uterus, firmly tied down to the neighboring organs, but with no breach of continuity in its mucous surfaces that I could detect. There was no ulcer; there was no fungus, no polypus, no fibroid. In less than a year her family followed her to the church-yard—because of *carcinoma uteri.*

Upon the question of menstruation: After listening to her recital in the parlor, as I have described it, how easily could I have written down the case in the precise language of Dr. Atlee's correspondent:

"In fact, she is at this time menstruating. When I say regular, I mean, of course, that in its fullest sense; she is regular as to time, quantity and quality, etc., and free from any abnormal symptoms."

Is this menstruation? Shall a woman of seventy, of eighty, mayhap of ninety-three years, having long passed the menopause, go through a sort of *senile puberty*, and take a new lease of menstrual life? If we should hold firmly the doctrine that the initiatory impulse of the menses proceeds from the ovaries, does it necessarily follow that the outward sign of this function must cease at once upon the withdrawal of its moving cause? Is it matter of great surprise that, in exceptional cases, the *sign* should recur once, twice, or even many times?

What is the teaching of nature on this point? How is it at the climacteric? Do we not all know that a more or less prolonged interval usually occurs between the commencing irregularity and the final cessation—called by Tilt the "dodging time"? Indeed, does not nature generally abhor any sudden and violent change in her economy?

May we not draw a lesson, too, from mechanics? What of a body moving responsively to a propelling force? If the force be suddenly withdrawn, do we expect the moving body to come instantly to a state of rest? Do we not know that it continues its motion for a time, in spite of the counter forces of friction and atmospheric resistance, by reason of the acquired momentum? And is there no analogue to momentum in the forces which move the nervous mass in man? If we suddenly decapitate an animal, do we expect to see the evidences of animal life instantly and wholly to disappear? Does anybody doubt for a moment that, in the higher order of animals, the great encephalic organs preside absolutely over the phenomena of life? Does anybody doubt that the decapitated animal is virtually dead?

How is it with traumatic epilepsy from depressed portion of skull? Suppose a length of time has elapsed, and the convulsive habit has been fully established. We now apply the trephine and remove the offending cause; are we greatly surprised if the effect (i. e., the epilepsy) should fail to disappear at once?

How about eclampsia? Do we not all know how much easier it is to control the first paroxysm than the second, and the second than the third? Is there not something like *momentum* in this? Does not hysteria, too, acquire increasing momentum under the influence of the same continued cause?

Take the case before us. We have seen that while this patient had formerly no menstrual flow from the uterus, speedily after the removal of the ovaries, a flow appears; and this, too, recurs several times subsequently, at irregular intervals—sometimes with abnormal nervous manifestations, sometimes without them. Is this menstruation? Shall we say that the presence of the ovaries *caused* the amenorrhœa, and their removal *cured* it, and *established true menstruation?* May not the moving force of ovulation, continued through ten, fifteen, or even twenty years, communicate to the nervous mass a *momentum* which shall continue to manifest itself, in exceptional cases, long after the ovaric force is withdrawn?

What say you then, my brethren? Will the removal of the ovaries determine the change of life?

I must crave your indulgence for having detained you so long upon this branch of the subject. I have purposely abstained from entering into the details of the physiology of menstruation, which could but weary your patience. I have refrained from the production of the very numerous authorities outside my own meagre library, and yet I have greatly desired that your convictions should be wholly unclouded upon this point.

If I have not effected *the change of life* by my interposition, then I have done nothing—worse than nothing—and it is in vain that I stand before you to-day. I am persuaded in my own mind that there is not one of you, my brethren, who will be disposed to take issue with me here, and I proceed, therefore, to consider the final question:

*Was it justifiable to effect a cure at the sacrifice of the ovaries?*

In the absence of any accumulated array of facts, it has been generally believed that the removal of the ovaries involved (*a*) danger to life; (*b*) loss of procreative power; (*c*) loss of the aphrodisiacal sense; (*d*) loss of menstrual molimen; (*e*) loss of the outward feminine graces—including mammary atrophy, change of voice, growth of beard, etc.

The fact that no surgical operation (however trivial in its nature), involving the use of the knife, is without danger to life, can not be too deeply impressed upon our minds. I shall not contend for a moment that the operation in question is free of danger; but I shall endeavor to satisfy you, by authorities, and by the results of observation and analogy, that the danger is not out of proportion to the severity of the malady, upon the one hand, nor the magnitude of the results upon the other.

The danger to life is of a three-fold character: *First*, that attending the simple incision, which is common to all operations, and which is trivial in importance, and therefore need not detain us here; *secondly*, the dangers attendant upon the opening of the peritoneal cavity; and *thirdly*, those consequent upon the removal of the ovaries.

*The opening of the peritoneal cavity* is certainly no new proceeding. There are very many instances already upon record where this has been done for various purposes, to say nothing of the long array of cases of operations for hernia and the modern ovariotomies. But to form a fair estimate of its dangers, we must divest the simple operation itself of all the complicating procedures of which it has been only the prelude. This, happily, we may do, in an appropriate way, by drawing our inferences from the exploratory incision for diagnosis in ovarian tumor.

After drawing a proper distinction between this procedure and an unfinished attempt at ovariotomy, in which more or less violence has been done to the abdominal contents, Dr. Peaslee remarks:

"Still less dangerous, however, is the making a short incision * * * for the express purpose of introducing the finger, or the steel sound, to ascertain the character of the tumor and the presence or absence of adhesions,—provided, always, that the patient's general health is not too much depressed. Of Dr. W. L. Atlee's two hundred and twenty-two collected cases, twenty-five were cases in which explorative incisions merely were made, and *all these recovered.* Dr. F. Bird stated, before the Medico-Chirurgical Society, November 12th, 1850, that he had made such incisions in between forty and fifty cases without any injurious consequences ensuing."*

I may remark, in passing, that I have had but one such case in my practice, in which I passed my fingers freely about in the cavity and down into the pelvis for the purposes of diagnosis. I had no reason, at any time, to regard the life of my patient in a particle of immediate danger.

While, as far as I am informed, there is no controversy upon the point of originality in the operation of normal ovariotomy, as I have defined it, there are a number of instances of more or less authenticity upon record in which the healthy ovaries have been successfully extirpated, or are supposed to have been removed. These cases, however, with exception of the one

* Ovarian Tumors: Peaslee. p. 177.

already cited from Percival Pott, are too vague and apocryphal to be here considered. They may be found in Dr. Peaslee's work, at page 226.

Allusion is also made in *Appleton's Journal*, of September 21, 1872, to the same operation, practiced among the Skopts, a religious sect in Roumania, as a religious rite, as circumcision is among the Jews. It appears that a fatal case had aroused the authorities to take cognizance of their doings; and as they are represented to have long practiced this rite, it would seem that it had been attended, probably, with no great degree of fatality, or attention would have been earlier drawn to the subject.

In the absence of statistics to guide us to a judgment of the fatality likely to ensue from normal ovariotomy, we can only approximate it by analogy.

It is a familiar fact that a like operation has been in practice, from time immemorial, upon the domestic animals. It is well known that the danger therefrom is exceedingly small; and this without any special care or nursing.

Of the statistics of ovariotomy, it is generally conceded that the rate of mortality, in the hands of experienced operators, is steadily decreasing. Upon this point Peaslee says:

"Ovariotomy, in well-selected cases, *is not comparatively a dangerous operation*, ninety per cent. recovering, probably, in the hands of experienced operators. Over eighty per cent. have actually been saved of cases not selected for their promising features, but taken as they came under the operator's notice, who rejected none which seemed to afford any chance of recovery from the operation."*

He likewise quotes Spencer Wells' opinion to similar purport: That short incisions through the abdominal parieties,—that the removal of a simple, unilocular cyst, and the absence of adhesions,—are all circumstances which materially diminish the mortality of ovariotomy, we would naturally expect; and such is the experience of operators and the dictum of the authorities.

Have we not in normal ovariotomy yet more favorable conditions than these; and may we not reasonably expect a yet greater reduction of mortality than has been attained, or is even attainable, in the ordinary operation of ovariotomy? If the

*Ovarian Tumors. p. 339.

opinion recently expressed by Dr. Marion Sims—that the mass of all the deaths from ovariotomy is caused by septicæmia, consequent upon the decomposition of bloody serum poured out within the cavity—shall prove to be correct, it is evident that the danger from this source is materially lessened in normal ovariotomy.

*The loss of possible power to perpetuate the species* is undeniable. But let me ask, Of what value is this function to a single lady of thirty, all her life an invalid, and hopelessly incurable excepting by the change of life, which itself implies the loss of this function? Indeed, we need not rest the case here; for there is abundant authority for the assertion that my patient, by reason of her endometritis and amenorrhœa, was, in all probability, *already sterile.*

Thomas says:

"An abnormal shape of the cervix has been pointed out by Dr. Sims as a frequent cause of infecundity. * * * My own experience leads me to the conclusion that, *excepting endometritis*, this is the *most common* of all the causes," etc. "Endometritis, whether it be cervical or corporeal, fills the uterine canal with a thick, tenacious mucus, which often prevents the entrance of seminal fluid."*

Byford says:

"Amenorrhœa is the least frequent of menstrual deviations as the effect of inflammation in the cervix uteri; but this inflammation is frequently the cause of scanty menstruation. It is curious to notice the manner in which this scantiness occurs. It seems to come on after the inflammation has lasted for a considerable time, and is *almost always associated with sterility.*"†

In a paper read before the Obstetrical Society of Berlin, by Dr. Carl Mayer,‡ upon the morbid conditions which give rise to sterility in women, in speaking of *chronic endometritis* as one of the conditions, he characterizes it as one of the *most obstinate* varieties of uterine disease, and asserts that he "*never saw a woman laboring under it become pregnant.*"

I venture the opinion, based upon my own limited observation, that conception coexisting with amenorrhœa *from any*

* Diseases of Women. p. 509.

† Medical and Surgical Treatment of Women. p. 160.

‡ American Journal of Medical Science, January, 1859. p. 269.

*cause*, of fifteen years' duration, is *very exceptional* in its occurrence. I have never known such an instance. I doubt whether any medical gentleman who hears me can recall *one* such case under his own observation. And yet I do not for a moment doubt that such exceptional case might occur, and has occurred; I only contend that they are exceptional—*very exceptional*—and do not vitiate the rule that long-standing amenorrhœa, from any cause, is eminently productive of sterility.

